# Lipoproteins as Drug Carriers for Cyclosporine A: Optimization of the Entrapment

**DOI:** 10.3390/ma16031156

**Published:** 2023-01-29

**Authors:** Mona M. A. Abdel-Mottaleb, Lorenza Boi, Marina Barra, Julie Colin, Luisa Berni, Arnaud Béduneau, Brice Moulari, Yann Pellequer

**Affiliations:** 1 Department of Pharmaceutics and Industrial Pharmacy, Ain Shams University, Cairo 11566, Egypt; 2 Department of Drug Science and Technology, University of Turin, 10125 Turin, Italy; 3 PEPITE EA4267, Labex LipSTIC (ANR-11-LABX-0021), Université Franche-Comté, F-25000 Besançon, France

**Keywords:** lipoproteins, cyclosporine A, cholesterol, entrapment, apolipoproteins

## Abstract

Lipoproteins are natural nanostructures responsible for the transport of cholesterol and other lipids in the blood. They are characterized by having a lipophilic core surrounded by an amphiphilic shell composed of phospholipids, cholesterol and one or more apolipoproteins. Being endogenous carriers makes them suitable for drug delivery purposes. Here, we investigate the effect of lipoproteins’ intricate composition on the entrapment efficiency of a model drug “Cyclosporine A” into the different types of lipoproteins, namely, HDL, LDL and VLDL. It was observed that the protein content of the lipoproteins had the highest effect on the entrapment of the drug with a correlation coefficient of 0.80, 0.81 and 0.96 for HDL, LDL and VLDL respectively. This was even confirmed by the effect of plasma on the association rate of lipoproteins and the drug. The second effective factor is the cholesterol concentration, while triglycerides and phospholipids had a negligible effect.

## 1. Introduction

Cyclosporine A (CsA) is an immunosuppressant drug widely used in organ transplant recipients and patients with autoimmune disorders [1] with a narrow therapeutic index [2]. The drug is also used with certain autoimmune diseases such as Behcet’s disease and rheumatoid arthritis [3]. It is known to be extensively bound to plasma and blood components (approximately 98%) leading to great variations in the free CsA concentration between different patients [2]. In plasma, about 98% is bound to proteins, 85–90% to lipoproteins and 5–15% to other proteins. In addition, when in whole blood, about 58–60% is found to bind to erythrocytes. CsA is an extremely lipophilic molecule with an octanol–water partition coefficient of 991 [2] which makes it highly entrapped in lipophilic drug delivery systems and facilitates a solubility of more than 500 mg/g in organic solvents such as methanol, ethanol and acetonitrile.

During the past few years, nanosystems have gained an interesting role as innovative drug delivery systems. They can be used to enhance drugs’ solubility, bioavailability and stability and increase targeting potential, enabling sustained effects with a reduction in side effects [4,5]. Lipoproteins are known to be a heterogeneous mixture of macromolecular aggregates of proteins and lipids responsible for the transport of lipids and lipophilic molecules from their sites of synthesis or absorption to the peripheral tissues within the systemic circulation [6]. They are described as spherical particles consisting of a nonpolar lipid core stabilized by a surface layer of amphipathic lipids such as phospholipids and unesterified cholesterol in addition to a special type of proteins called apolipoproteins [7]. Because proteins are in general considered more dense than lipids, lipoproteins with a higher protein-to-lipid ratio are of a higher density compared to the ones containing a higher lipid content. Hence, lipoproteins are classified according to their density into five main categories, namely, chylomicrons, very low density lipoproteins (VLDL), intermediate-density lipoproteins (IDL), low-density lipoproteins (LDL), and high-density lipoproteins (HDL). In terms of particle size, chylomicrons are considered the largest lipoproteins (100–1000 nm) followed by VLDL (30–80 nm), both being rich in triglyceride content. The product of VLDL metabolism is IDL, which is more rich in CE and is then metabolised to LDL (18–25 nm), in which almost all the TG content is hydrolysed and the core is mainly composed of CE. The smallest type of lipoproteins is HDL (7–12 nm) [6]. Lipoproteins have recently been proposed as drug delivery carriers because of their endogenous nature that makes them naturally biocompatible, non-immunogenic and have the potential to escape recognition by the reticuloendothelial system [8,9]. In addition, they can be used as targeting systems, for example, LDL is mainly cleared via specific LDL receptors in the liver. In addition, some types of tumours show a very high rate of LDL uptake. The oily core of lipoproteins provides an ideal carrier for many lipophilic drugs [10]. Drugs that are transported in the core of lipoproteins are protected from the environment during transport, and the environment is protected from the drug until it reaches the targeted site. Several studies have described the use of different lipoproteins for drug delivery purposes in different diseases [8,11,12]. As cyclosporine has been reported to interact with plasma lipoproteins to an extent that affects its pharmacokinetics and toxicity, it was chosen here as a model drug to be associated with the different types of lipoproteins, namely, HDL, LDL and VLDL. The effect of the detailed composition of the different lipoproteins on the entrapment efficiency of the drug was explored in order to provide a clear understanding of the possible ways to enhance the entrapment into lipoproteins for drug delivery purposes.

## 2. Materials and Methods

### 2.1. Materials

Plasma from human donors was purchased from the centre of blood donation Etablissement Francais du Sang; EFS Besancon. Samples were obtained from 10 different donors, and each sample was used for the preparation of the three types of lipoproteins. Cyclosporine A and bovine serum albumin (BSA) were purchased from Sigma Aldrich in France (St. Quentin Fallavier). Potassium bromide was bought from Acros Organics in Belgium (Geel). Tubes for ultracentrifugation, Bottle with Cap Assembly, Polycarbonate, 26.3 mL, 25 × 89 mm were obtained from Beckman, UK (High Wycombe). Amicon Ultra 30 K Centrifugal Filter Devices were purchased from Merck Millipore in Germany (Darmstadt). Assay kits for the determination of total cholesterol, free cholesterol and triglycerides were obtained from DiaSys diagnostics, France (Grabels). BCA and micro-BCA assay kits for the determination of protein concentrations were purchased from Pierce, Fischer-Thermo Scientific, France (Artenay).

### 2.2. Methods

#### 2.2.1. Preparation of Plasma or Lipoproteins

The separation of the lipoproteins was performed using sequential ultracentrifugation as previously described elsewhere [13]. Put simply, VLDL were separated from human plasmas through ultracentrifugation as a fraction less than or equal to 1.006 after 20 h of centrifugation at 45,000 rpm. An LDL fraction of 1.019 < d < 1.063 g/mL density was isolated with one 22 h, 50,000 rpm in a 70 Ti rotor in an Optima L90-K ultracentrifuge (Beckman, Brea, CA, USA). The HDL were ultracentrifugally isolated from the plasmas as a 1.070 < d < 1.210 g/mL density fraction, with one 24 h, 50,000 rpm spin. The densities were adjusted with the addition of KBr [14]. The isolated lipoproteins were recovered and concentrated through centrifugation with the 5810 R Eppendorf centrifuge (Eppendorf SE, Hamburg, Germany) into Amicon Ultra 30 K Centrifugal Filter Devices at 4000 rpm for 45 min and stored at 4 °C after being passed under a stream of nitrogen to avoid lipoperoxidation.

#### 2.2.2. Incubation of Cyclosporine A with Plasma or Lipoproteins

For cyclosporine loading into the lipoproteins, two techniques were performed. The first technique was to directly incubate the already isolated lipoproteins, namely, VLDL, LDL and HDL, with excess cyclosporine A for 6 h at 37 °C under agitation. At the end of the incubation, the tubes were centrifuged for 1 min at 1000 rpm to remove the excess un-dissolved cyclosporine A. The supernatant was ultra-filtered with Amicon Ultra 30 K Centrifugal Filter Devices through centrifugation for 10 min at 14,000 rpm, 4 °C, and the filtrate was recovered with a second cycle at 1000 rpm, 5 min, 4 °C, placing the filter upside-down. The recovered lipoproteins were then diluted with PBS to be analysed for cyclosporine A content. The second technique was to incubate the full plasma with excess cyclosporine A at 37 °C for 6 h under agitation. Each type of lipoproteins was then separated as described above and the drug content analysed to evaluate the yield of association between each type of lipoproteins and the drug.

The samples of lipoproteins were extracted using acetonitrile that was filtered through 0.22 µm membrane filters and injected into HPLC 1220 Infinity (Agilent, Santa Clara, CA, USA) equipped with 5 µ C18 Klinetex column at 60 °C. The mobile phase was formed of acetonitrile and water with proportions changing in a gradient flow according to Table 1. The results of the encapsulated cyclosporine were normalized to the volume of 1 L plasma to facilitate comparisons.

#### 2.2.3. Protein and Lipid Analyses

All chemical assays for the determination of cholesterol content and phospholipids and triglycerides concentrations in the lipoproteins were performed using assay kits for the determination of total cholesterol, free cholesterol, and phospholipids and triglycerides obtained from DiaSys diagnostics, France, according to the manufacturer’s instructions. The BCA and micro-BCA assay kits for the determination of the protein concentrations, based on the principle explained by Smith et al. [15], were purchased from Pierce, Fischer-Thermo Scientific.

## 3. Results

**Figure 1:**
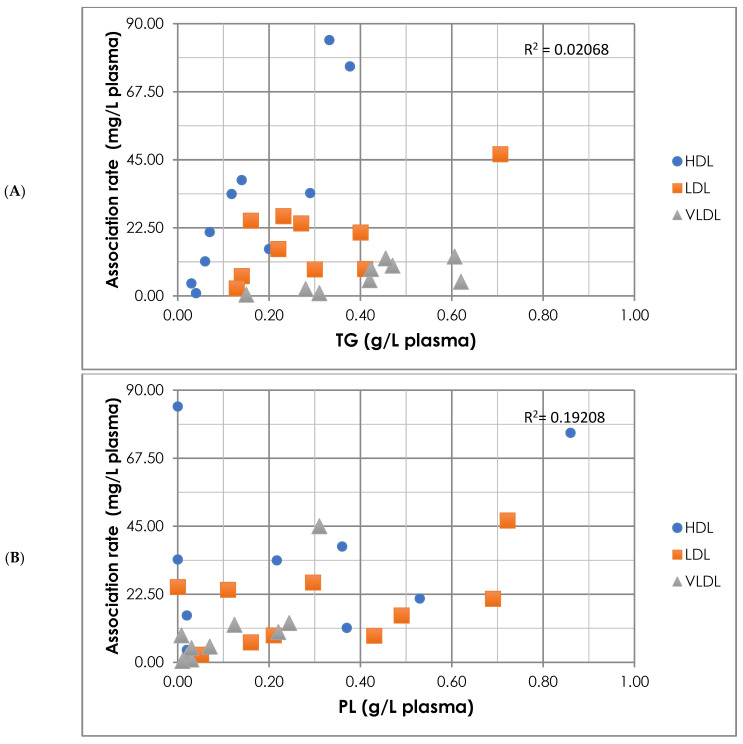

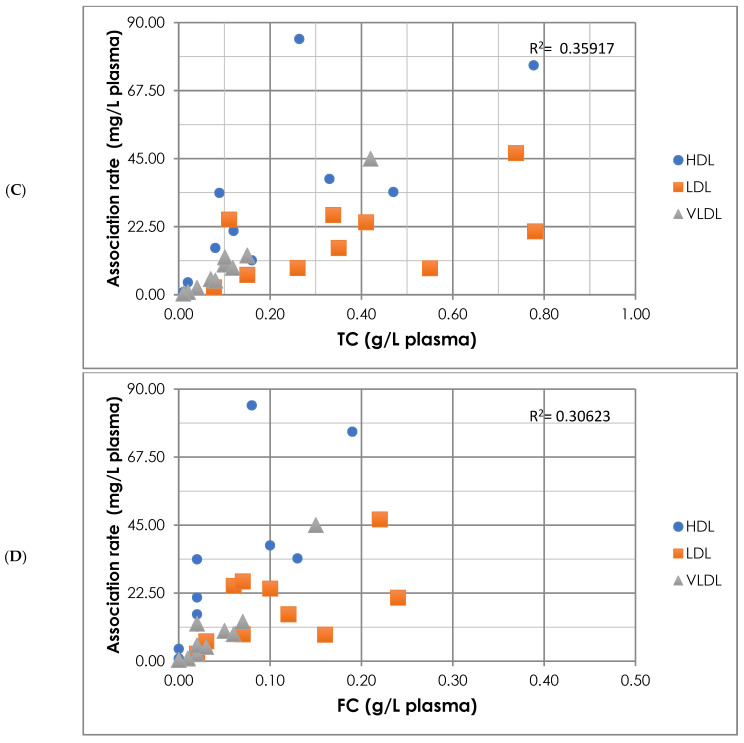

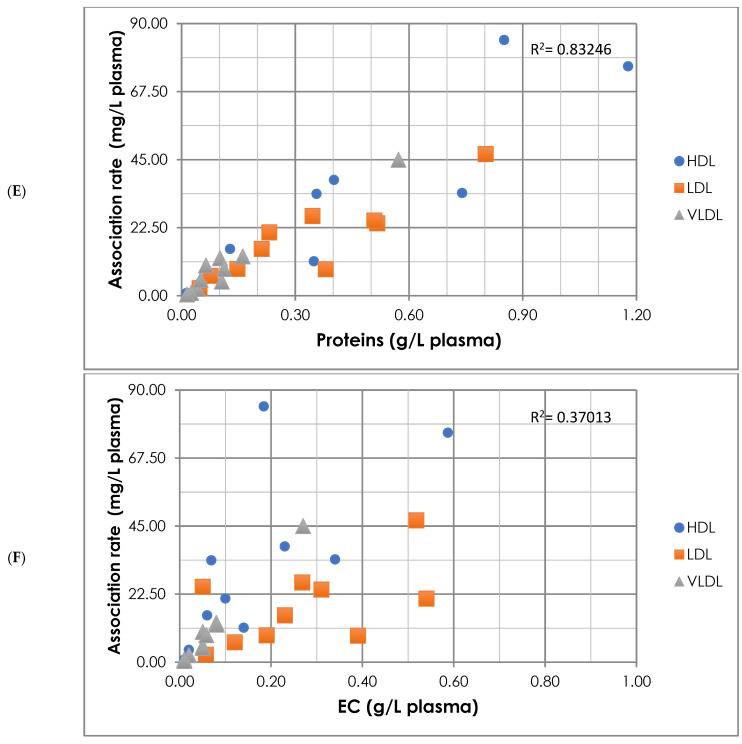
A correlation between the concentration of lipoprotein components and the cyclosporine association rate; (**A**): triglyceride concentration, (**B**): phospholipids concentration, (**C**): total cholesterol concentration, (**D**): free cholesterol concentration, (**E**): proteins concentration, (**F**): esterified cholesterol concentration.

CycloA is a hydrophobic drug that can interact with the lipid components of lipoproteins as well as the proteins on their surface. The biochemical composition of all the types of lipoproteins was studied in order to find a correlation with the association rate of CycloA. This biochemical analysis included studying the concentrations of triglycerides (TG), total cholesterol (TC), free cholesterol (FC), esterified cholesterol (EC = TC − FC), phospholipids (PL) and proteins and was carried out before incubation of each type of the lipoproteins with CycloA. The obtained data were normalized to 1 L of plasma to facilitate comparison. The rates of CycloA association were then compared and related to the composition of the lipoproteins. A correlation between the association rate of cyclosporine A with the different lipoproteins and the concentration of the individual components of the lipoproteins is expressed in Figure 1 and Supplementary Table S1. The highest correlation was found to be associated with the protein concentration in the lipoproteins (0.83). The triglycerides and cholesterol levels, either free or esterified, follow; then, the effect of phospholipids was found to be very limited. In addition, a specific correlation was performed for each type of lipoproteins independently, and the results are stated in Table 2.

**Figure 2:**
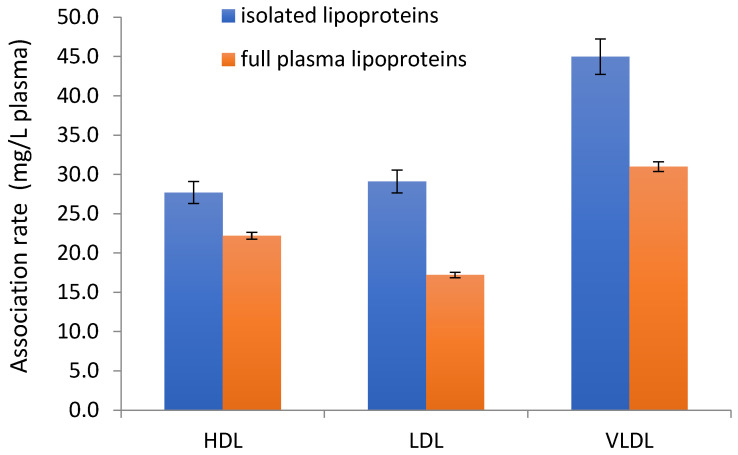
Difference in the association rate of cyclosporine with the different types of lipoproteins before and after separation from the plasma.

To evaluate the influence of the proteins and enzymes present in the full plasma that can promote or prevent the association of CycloA in the lipoproteins, a comparison was carried out between the associations of CycloA with isolated lipoproteins and with lipoproteins in full plasma obtained from the same donor. Detailed data on the difference between the isolated lipoproteins and full plasma incubation are shown in Table 3 and Figure 2. Generally, there was lower encapsulation in the lipoproteins when mixed with the drug in the form of full plasma compared to the previously isolated form.

## 4. Discussion

In order to use lipoproteins as a drug carrier, it is necessary to understand the influence of their intricate composition on the ability to interact with or encapsulate the drug. In our study, it was found that the interaction between CsA and the lipoproteins was mainly dependent on their protein content. Cyclosporine is a lipophilic molecule; in blood, around 40% is bound to erythrocytes, while most of the rest is bound to lipoproteins. In general, CsA is primarily transported bound to lipoproteins (33%) containing cholesterol, triglycerides, phospholipids and proteins. The HDL and LDL fractions bind around 80% or more of the bound CsA, while the amount bound to VLDL is much less, and it is also known that whole blood CsA levels correlate with lipoprotein levels [1]. In vitro and in vivo studies reported that in healthy patients, analyses of CsA behaviour revealed that 50–60% of CsA is bound to HDL, 20–30% to LDL, 10–25% to VLDL and 10–15% to the non-lipoprotein proteins [16,17]. However, the proportion of CsA bound to the LDL and VLDL fractions was higher in hyperlipidaemic patients, without changing the amount bound to free protein, indicating that the distribution of CsA between the lipoprotein classes changes as plasma lipoprotein concentrations or composition change. A more detailed study found that the plasma distribution of CsA in the different lipoproteins followed this order: HDL > LDL > VLDL > non-lipoprotein proteins. The correlation between the CsA concentration added and its association with different lipoproteins followed a linear pattern up to the concentration of 100 pmol/mL serum, but at 200 pmol/mL, VLDL and LDL started to show a saturation pattern while HDL continued to show a linear correlation, indicating the importance of its higher protein content in the binding of CsA to the lipoproteins [17]. The binding of CsA to lipoproteins led to reduced uptake of the drug by hepatocytes. In the absence of lipoproteins, CsA was rapidly taken up by isolated rat hepatocytes, and up to 80% was associated with the cells. However, in the presence of LDL and HDL, a significant reduction in CsA uptake and metabolism was noticed, and this reduction was related to the concentration of lipoproteins added [18]. The strong interaction with lipoproteins has been also proven in a perfused rat kidney model, where renal elimination was significantly reduced by the addition of HDL or LDL to the medium [19].

In a comparative study of CsA entrapment into liposomes and lipoproteins, it was found that 40–60% of the CsA was easily displaced from the liposomes, while this was not observed in the case of the lipoproteins. This suggests that proteins present in the lipoproteins are involved in the uptake of CsA and strongly involved in the drug’s association with the lipoproteins [17].

Another example of the effect of lipoprotein composition on the association with CsA is the finding that the high triglyceride content of HDL was associated with a decreased percentage of CsA recovered in the HDL fraction and an increased percentage recovered in the VLDL fraction [20]. In another reported case of intralipid infusion, the VLDL fraction rose to 56% and the levels of HDL and LDL significantly fell, leading to a sudden rise in the free, unbound CsA concentration in the blood, which then accumulated in the brain. The high accumulation in the brain was due to the presence of high concentrations of the protein cyclophilin, giving more proof of the importance of protein to bind CsA [21].

As previously mentioned, CsA is highly bound to erythrocytes in the blood as well. In a trial to test for the exact factors affecting this finding, it was found that the presence of both albumin and plasma in the medium retarded the uptake of cyclosporine into the red blood cells but did not influence drug efflux out of the cells. The effects of albumin were proportional to the concentration of albumin present, whereas the plasma samples taken after the ingestion of food led to a more pronounced decrease in cyclosporine uptake, possibly because of the presence of chylomicrons in plasma [2]. The effect of albumin on drug–erythrocyte binding gives a clear explanation of the reduced binding of CsA in full plasma lipoproteins in our results compared to the isolated lipoproteins. Shibata et al. found that almost all CsA bound to erythrocytes was found to bind to a specific protein called cyclophillin, confirming the high affinity of CsA to proteins in general [22].

From the above, the use of lipoproteins for CsA delivery is an interesting strategy to provide the required solubility, stability and potential targeting of the drug. The interaction of CsA with lipoproteins is considered strong enough that the drug remains inside the particles even after ultracentrifugation yet loose enough to allow in vitro transfer between the various types of lipoproteins and in vivo transfer to the tissues. The concentration of CsA required to produce a 50% inhibition in the proliferative activity of T Lymphocytes (IC50) was found to be significantly reduced in the presence of different lipoproteins. Only 189 and 80 µg/L were required in the presence of VLDL and LDL, respectively, compared to 300 µg/L of the free CsA. It was claimed that the formation of an LDL–CsA complex could potentiate the immunosuppressive activity by increased uptake through LDL receptors [23].

The second effective factor in the association of CsA to lipoproteins after protein concentration is the cholesterol concentration. The free, unbound CsA concentration was found to be significantly increased with subsequently increased clearance after the administration of lipid-lowering drugs like simvastatin [24]. Another study assumed that because CsA associates with lipoproteins in the plasma after administrating, then the composition and concentration of lipoproteins would have a significant effect on its distribution. It was concluded that CsA lipoprotein distribution is partially affected by the cholesterol concentration and to a lesser extent by the triglyceride concentration [6]. Patients with low cholesterol levels in the blood were shown to have a higher incidence of CNS and neurotoxicity. This is due to the higher concentration of unbound, free CsA in the blood, indicating a kind of tendency of CsA to bind with cholesterol [25]. In addition, patients with high total plasma cholesterol levels were found to show increased CsA association with plasma LDL compared to patients with a normal blood lipid profile [26].

The clinical application of cyclosporine loaded into lipoproteins is beneficial in various situations. Cyclosporine A is a potent immunosuppressant with a low safety profile. Its use in various organ transplantations and the treatment of autoimmune diseases such as rheumatoid arthritis and ulcerative colitis has been reported to be successful [27]. Although the use of intravenous cyclosporine has proven effective in most cases of refractory ulcerative colitis, the systemic side effects like nephrotoxicity, hypertension, seizures and opportunistic infections have been reported, especially in patients with low plasma lipid concentrations [28,29]. The interesting affinity of cyclosporine A to the different lipoproteins and especially LDL offers a great potential to use the lipoproteins as a biocompatible drug delivery carrier. Lipoproteins are considered natural nanocarriers with a lipophilic core containing cholesterol esters surrounded by a shell of amphiphilic phospholipids and apoproteins. Their particle size ranges from 1200 nm for chylomicrons to 8–12 nm in the case of HDL [7,8]. Being natural components of the blood, they are hardly recognized by the immune system, especially the in case of using lipoproteins from the patient for the treatment of certain diseases. In addition, it has been reported that LDL receptors are upregulated in cancerous tissues and inflammatory diseases where there is a higher need to synthesize new cell membranes. This opens the potential for using lipoproteins loaded with different drugs for targeting certain diseases such as ulcerative colitis, especially using LDL [9].

## 5. Conclusions

In the current study, cyclosporine A was encapsulated into different types of lipoproteins. The effect of the composition of the lipoproteins on the association efficiency was evaluated. It was observed that the protein content of the lipoproteins had the highest effect on the entrapment of the drug with a correlation coefficient of 0.80, 0.81 and 0.96 for HDL, LDL and VLDL, respectively. This was even confirmed by the effect of plasma on the association rate of lipoproteins and the drug. The second effective factor is the cholesterol concentration, while triglycerides and phospholipids had a negligible effect.

## Figures and Tables

**Table 1:** Percentage of solvents (water and acetonitrile) during the gradient flow analysis of cyclosporine A using HPLC.

Time (min)	ACN (%)	H_2_O (%)
0	68	32
2	68	32
5	85	15
5.1	95	5
7.1	95	5
7.2	68	32
10.2	68	32

**Table 2:** Effect of lipoprotein type and composition on the association efficiency of cyclosporine A.

Lipoprotein	Correlation Coefficient Between LPs’ Different Components and Association Efficiency					
	TG	PL	TC	FC	Proteins	EC
HDL	0.7513	0.1196	0.5134	0.5610	0.8138	0.4889
LDL	0.3721	0.2337	0.3392	0.3079	0.8090	0.3386
VLDL	0.9325	0.6459	0.9830	0.8854	0.9608	0.9875

**Table 3:** Difference in the association rate of cyclosporine with the different types of lipoproteins before and after separation from the plasma.

Carrier	Type of Lipoproteins	TG (g/L Plasma)	PL (g/L Plasma)	TC (g/L Plasma)	FC (g/L Plasma)	Proteins (g/L Plasma)	EC (g/L Plasma)	Encapsulation Rate (mg/L Plasma)
isolated LPs	HDL	0.120	0.160	0.080	0.01	0.342	0.070	27.700
	LDL	0.970	0.370	0.52	0.12	0.566	0.400	29.100
	VLDL	2.890	0.310	0.420	0.15	0.572	0.270	45.000
full Plasma	HDL	0.290	0.230	0.150	0.04	0.673	0.110	22.200
	LDL	1.620	0.360	0.630	0.14	0.774	0.490	17.200
	VLDL	3.800	0.250	0.470	0.16	0.741	0.310	31.000

## Data Availability

Not applicable.

## Supplementary Materials

The following supporting information can be downloaded at: https://www.mdpi.com/article/10.3390/ma16031156/s1, Supplementary Table S1: The detailed composition of the different lipoproteins and the corresponding association rate with Cyclosporine A.
