# Supplementary material for: Lipoproteins as Drug Carriers for Cyclosporine A: Optimization of the Entrapment
Source: Materials (Basel). 2023 Jan 29;16(3):1156. doi: 10.3390/ma16031156 (PMC9918909; doi:10.3390/ma16031156)
Supplement: Supplementary file 1 [file materials-16-01156-s001.zip › materials-2074046-supplementary.pdf]

Donor	Type of Lipoproteins	TG (g/L plasma)	PL (g/L plasma)	TC (g/L plasma)	FC (g/L plasma)	Proteins (g/L plasma)	EC (g/L plasma)	Encapsulation rate (mg/L plasma)
**1**	HDL	0.200	0.020	0.080	0.020	0.128	0.060	15.500
	LDL	0.400	0.690	0.780	0.240	0.231	0.540	21.000
	VLDL	0.280	0.020	0.040	0.020	0.042	0.020	2.300
**2**	HDL	0.290	0.000	0.470	0.130	0.740	0.340	34.000
	LDL	0.300	0.430	0.550	0.160	0.380	0.390	8.800
	VLDL	0.310	0.030	0.020	0.010	0.025	0.010	0.900
**3**	HDL	0.040	0.020	0.010	0.000	0.013	0.010	0.900
	LDL	0.410	0.210	0.260	0.070	0.147	0.190	8.900
	VLDL	0.420	0.070	0.070	0.020	0.050	0.050	5.200
**4**	HDL	0.060	0.370	0.160	0.020	0.349	0.140	11.400
	LDL	0.140	0.160	0.150	0.030	0.076	0.120	6.600
	VLDL	0.150	0.010	0.010	0.000	0.014	0.010	0.400
**5**	HDL	0.030	0.020	0.020	0.000	0.048	0.020	4.100
	LDL	0.270	0.110	0.410	0.100	0.516	0.310	24.000
	VLDL	0.620	0.030	0.080	0.030	0.106	0.050	4.700
**6**	HDL	0.140	0.360	0.330	0.100	0.402	0.230	38.300
	LDL	0.160	0.000	0.110	0.060	0.508	0.050	24.900
	VLDL	2.890	0.310	0.420	0.15	0.572	0.270	45.000
**7**	HDL	0.070	0.530	0.120	0.020	0.230	0.100	21.100
	LDL	0.220	0.490	0.350	0.120	0.211	0.230	15.500
	VLDL	0.470	0.220	0.100	0.050	0.064	0.050	10.000
**8**	HDL	0.377	0.860	0.777	0.190	1.178	0.587	75.900
	LDL	0.129	0.051	0.077	0.020	0.047	0.057	2.500
	VLDL	0.606	0.244	0.150	0.070	0.161	0.080	13.000
**9**	HDL	0.118	0.217	0.089	0.020	0.356	0.069	33.700
	LDL	0.231	0.296	0.338	0.070	0.345	0.268	26.400
	VLDL	0.455	0.124	0.101	0.020	0.101	0.081	12.400
**10**	HDL	0.332	0.000	0.264	0.080	0.851	0.184	84.600
	LDL	0.706	0.722	0.738	0.220	0.802	0.518	46.900
	VLDL	0.423	0.008	0.118	0.060	0.112	0.058	8.900

Supplementary Table S1: The detailed composition of the different lipoproteins and the corresponding association rate with Cyclosporine A.
